# Anti-Inflammatory Effects of Monoammonium Glycyrrhizinate on Lipopolysaccharide-Induced Acute Lung Injury in Mice through Regulating Nuclear Factor-Kappa B Signaling Pathway

**DOI:** 10.1155/2015/272474

**Published:** 2015-03-18

**Authors:** Xiaoying Huang, Jiangfeng Tang, Hui Cai, Yi Pan, Yicheng He, Caijun Dai, Ali Chen, Xiaoming Yu, Mayun Chen, Lizhen Zou, Liangxing Wang

**Affiliations:** ^1^Department of Respiratory, The First Affiliated Hospital of Wenzhou Medical University and Key Laboratory of Heart and Lung, Wenzhou, No. 2 Fu Xue Lane, Lucheng District, Wenzhou, Zhejiang 325035, China; ^2^Respiratory Department, Changxing Traditional Chinese Medicine Hospital, No. 198 Binnan Road, Changxing Town, Huzhou, Zhejiang 313100, China

## Abstract

The present study aimed to investigate the therapeutic effect of monoammonium glycyrrhizinate (MAG) on lipopolysaccharide- (LPS-) induced acute lung injury (ALI) in mice and possible mechanism. Acute lung injury was induced in BALB/c mice by intratracheal instillation of LPS, and MAG was injected intraperitoneally 1 h prior to LPS administration. After ALI, the histopathology of lungs, lung wet/dry weight ratio, protein concentration, and inflammatory cells in the bronchoalveolar lavage fluid (BALF) were determined. The levels of tumor necrosis factor-*α* (TNF-*α*) and interleukin-1*β* (IL-1*β*) in the BALF were measured by ELISA. The activation of NF-*κ*B p65 and I*κ*B-*α* of lung homogenate was detected by Western blot. Pretreatment with MAG attenuated lung histopathological damage induced by LPS and decreased lung wet/dry weight ratio and the concentrations of protein in BALF. At the same time, MAG reduced the number of inflammatory cells in lung and inhibited the production of TNF-*α* and IL-1*β* in BALF. Furthermore, we demonstrated that MAG suppressed activation of NF-*κ*B signaling pathway induced by LPS in lung. The results suggested that the therapeutic mechanism of MAG on ALI may be attributed to the inhibition of NF-*κ*B signaling pathway. Monoammonium glycyrrhizinate may be a potential therapeutic reagent for ALI.

## 1. Introduction

Acute lung injury (ALI) is a life-threatening illness syndrome characterized by widespread capillary leakage, low lung compliance, severe resistant hypoxemia, severe difficulty breathing, increased rate of breathing, and even multiple organ failure. The pathophysiology of ALI shows as alveolar-capillary barrier damage with noncardiogenic pulmonary edema, excessive invasion of inflammatory cells, and release of inflammatory mediators [1, 2]. The incidence of acute lung injury was 78.9 per 100,000 person-years and the age-adjusted incidence was 86.2 per 100,000 person-years [3]. Acute lung injury (ALI) and its more severe form, acute respiratory distress syndrome, (ARDS) have mortality rates of 40–70% caused by direct injury, such as pneumonia or aspiration, and indirect injury, such as sepsis or trauma [3, 4]. Infection is the most common cause of ALI clinically. Lipopolysaccharide (LPS) is a main component of the gram-negative bacterial cell wall, which could activate Toll like receptor (TLR) 4 and trigger an inflammatory response, including recruit polymorphonuclear leukocytes (PMNs) into the lung with increasing in capillary permeability [5]. Many methods can generate ALI. Intratracheal instillation of LPS has gained wide acceptance as a reliable model of severe lung injury [6]. Lipopolysaccharide administration can intratracheally induce the apoptosis of lung epithelial cell and rapid influx of inflammatory cells, releasing inflammatory cytokines, reactive oxygen species, and chemotactic factors. These effects cause the inflammation in alveolar space and injury alveolar barrier, ultimately resulting in acute lung injury [7, 8]. There are still few specific medicines to treat it. Monoammonium glycyrrhizinate (MAG), also named glycyrrhizinic acid monoammonium salt, is a bioactive plant component isolated from licorice root. Monoammonium glycyrrhizinate could transform to 18*β*-glycyrrhetinic acid [9]. 18*β*-glycyrrhetinic acid has various pharmacological actions such as anti-inflammatory, antiallergic, antigastric ulcer, and antihepatitis activities [10, 11]. Monoammonium glycyrrhizinate was found to reduce cytokine production and improve mortality in LPS-induced endotoxin shock in mice [12]. Monoammonium glycyrrhizinate also has been reported to have anti-inflammatory effect by regulating TNF-*α*/IL-10 balance [13]. However, there were limited reports about the effect of MAG on NF-*κ*B signaling pathway in LPS-induced acute lung injury. In this study, we investigated the effects of MAG on LPS-induced ALI and elucidated the potential mechanism: (1) pulmonary edema and protein in BALF, (2) histopathological changes and neutrophil infiltration, (3) and TNF-*α* and IL-1*β* production (4) especially NF-*κ*B activation.

## 2. Materials and Methods

### 2.1. Animal

In this study, BALB/c mice (male, 6–8 weeks old, and 20–25 g) were purchased from the SLAC Laboratory Animal Ltd., Co. (Shanghai, China). All experimental protocols were approved by the Ethical Committee on Animal Research at the Wenzhou Medical University. The mice were housed in a room with controlled temperature at 23 ± 2°C and relative humidity of 50% with 12 h light/dark cycle. All procedures were performed in accordance with the guide for the Care and Use of Laboratory Animals published by the US National Institute of Health.

### 2.2. Reagents

Monoammonium glycyrrhizinate (MAG) was purchased from Sigma Chemical Co. (St. Louis, MO, USA) and suspended in phosphate-buffered saline (PBS). Lipopolysaccharide (*Escherichia coli* 055:B5) was purchased from Sigma Chemical Co. (St. Louis, MO, USA). Mouse TNF-*α* and IL-1*β* enzyme linked immunosorbent assay (ELISA) kits were purchased from R&D (Minneapolis, MN, USA). Mouse mAb NF-*κ*B p65 and mouse mAb I*κ*B*α* were purchased from Cell Signaling Technology Inc. (Beverly, MA, USA). HRP-conjugated goat anti-rabbit antibody was provided by Beyotime (Haimen, China). The Nuclear and Cytoplasmic Protein Extraction Kit and BCA protein assay kit were provided by Thermo Scientific (Rockford, IL, USA).

### 2.3. LPS-Induced ALI in Mice

Mice were randomly divided into five groups: control group, LPS group, and LPS + MAG (3, 10, and 30 mg/kg) groups. Each group contained eight mice. Mice were anesthetized with intraperitoneal injection of sodium pentobarbital (50 mg/kg). Before inducing acute lung injury, the mice were given intraperitoneal injection with MAG (3, 10, and 30 mg/kg). One hour later, LPS (5 mg/kg) was instilled intratracheally to induce acute lung injury. Normal mice were given PBS. Twenty-four hours after LPS administration, lung tissues and BALF were collected.

### 2.4. Pulmonary Histopathology

The upper lobe of right lung was excised at 24 h after LPS administration. The lungs were fixed in 4% paraformaldehyde for 24 h at 4°C, then embedded in paraffin, and sliced into 4 *μ*m sections. Hematoxylin-eosin stains were performed using standard protocol. After staining, histopathological changes in the lung tissues were observed under a light microscope.

### 2.5. Bronchoalveolar Lavage

At 24 h after LPS challenge, a median sternotomy allowed exposing both of the lungs. The trachea was inserted with a 24G intravenous infusion needle. After ligating the hilum on the right lung, the left lung was lavaged three times with 0.4 mL ice-cold phosphate-buffered saline. The recovery ratio of the fluid was about 90% (nearly 1 mL). The bronchoalveolar lavage fluid (BALF) was immediately centrifuged at 500 g for 10 min at 4°C, and the cell-free supernatants were stored at −80°C for cytokine analysis and protein measurement. The cell pellet was resuspended in PBS for total cell counts using hemacytometer and the remaining cell samples were smeared on a slide, and the percentage of neutrophils was calculated by staining with the Wright-Giemsa staining method.

### 2.6. Lung Wet/Dry Weight Ratio and Protein Concentration in BALF

The ratio of the wet lung to the dry lung was an index of lung edema. The middle lobe of right lung was excised and the wet weight was recorded. The lobe was then placed in an incubator at 70°C for 24 h to obtain the dry weight. The lung wet/dry weight ratio was calculated to assess tissue edema. To evaluate vascular permeability in the lung, the protein concentration in BALF was quantified by BCA method.

### 2.7. Cytokine Assay

Concentrations of TNF-*α* and IL-1*β* in BALF were determined by ELISA kits according to the manufacturer's instructions. The optical density of each well was read at 450 nm.

### 2.8. Western Blot Analysis

Lung tissues samples were collected at 24 h after LPS administration and frozen in liquid nitrogen immediately until homogenization. Nuclear and cytoplasmic proteins were extracted using the Nuclear and Cytoplasmic Protein Extraction Kit. Nuclear protein extracts were used to detect the NF-*κ*B p65 subunit and Lamin B; cytoplasmic protein extracts were used to detect I*κ*B-*α* and GAPDH. Protein concentrations were determined with a bicinchoninic acid (BCA) protein assay kit. Proteins were separated on a 12% sodium dodecyl sulfate- (SDS-) polyacrylamide gel and transferred onto polyvinylidene difluoride (PVDF) membranes, and the membranes were blocked in 5% skim milk (Sigma) at room temperature for 1 h. The membranes were incubated at 4°C overnight with antibodies. Subsequently, the membranes were incubated with HRP-conjugated secondary antibody at room temperature for 1 h. The signal was visualized by enhanced chemiluminescence (ECL) reagents according to the manufacturer's protocol. Antibodies to Lamin B and GAPDH were used as internal controls of nuclear and cytosolic protein loading, respectively. All blotting experiments were performed at least three times with different mice.

### 2.9. Statistical Analysis

Data were entered into a database and analyzed using SPSS software. All values were expressed as mean ± SD. Differences among multiple groups were analyzed by one-way ANOVA and Student's* t*-test. Statistical significance was defined by a *P* < 0.05 (two-tailed).

## 3. Results

### 3.1. Effect of MAG on the Lung Edema and Protein Concentration in the BALF of Mice with ALI

**Figure 1:**
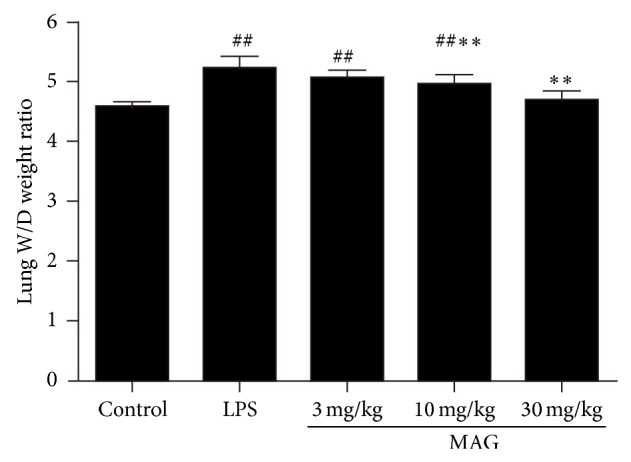
Effect of MAG on the lung W/D weight ratio of LPS-induced ALI mice. MAG (3, 10, and 30 mg/kg) was injected intraperitoneally 1 h prior to LPS instillation. The lung W/D weight ratio was determined at 24 h after LPS was given. The values presented are the mean ± SD. ^#^ *P* < 0.05, ^##^ *P* < 0.01 versus the control group; ^*^ *P* < 0.05, ^**^ *P* < 0.01 versus the LPS group.

**Figure 2:**
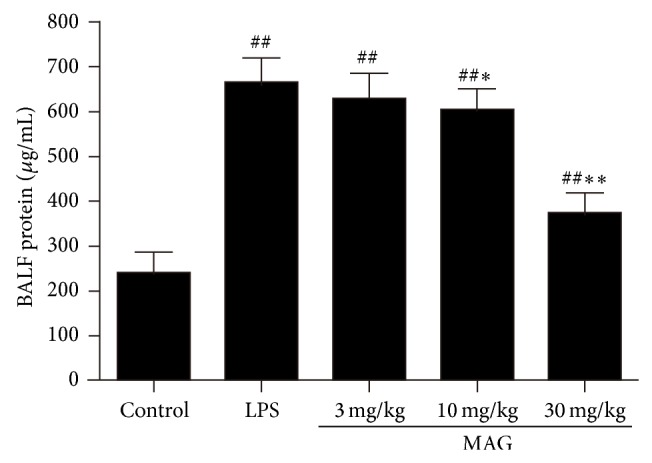
Effect of MAG on the total protein content in BALF of LPS-induced ALI mice. MAG (3, 10, and 30 mg/kg) was injected intraperitoneally 1 h prior to LPS instillation. The total protein content in BALF was determined at 24 h after LPS was given. The values presented are the mean ± SD. ^#^ *P* < 0.05, ^##^ *P* < 0.01 versus the control group; ^*^ *P* < 0.05, ^**^ *P* < 0.01 versus the LPS group.

The lung W/D weight ratios were significantly higher at 24 hours after LPS challenge, compared to the control group (*P* < 0.01) (Figure 1). The increase of the lung W/D weight ratios was significantly reduced by high and medium dose of MAG (10 and 30 mg/kg) administration (*P* < 0.01 and *P* < 0.01, resp.). MAG significantly reduced lung edema formation. We also examined total protein concentration in BALF. The result showed that the total protein concentration increased significantly in the LPS group (*P* < 0.01), and high and medium dose of MAG (10 and 30 mg/kg) depressed LPS-induced protein concentration (*P* < 0.05 and *P* < 0.01, resp.) (Figure 2).

### 3.2. Effect of MAG on the Pulmonary Histopathological Changes of Mice with ALI

**Figure 3:**
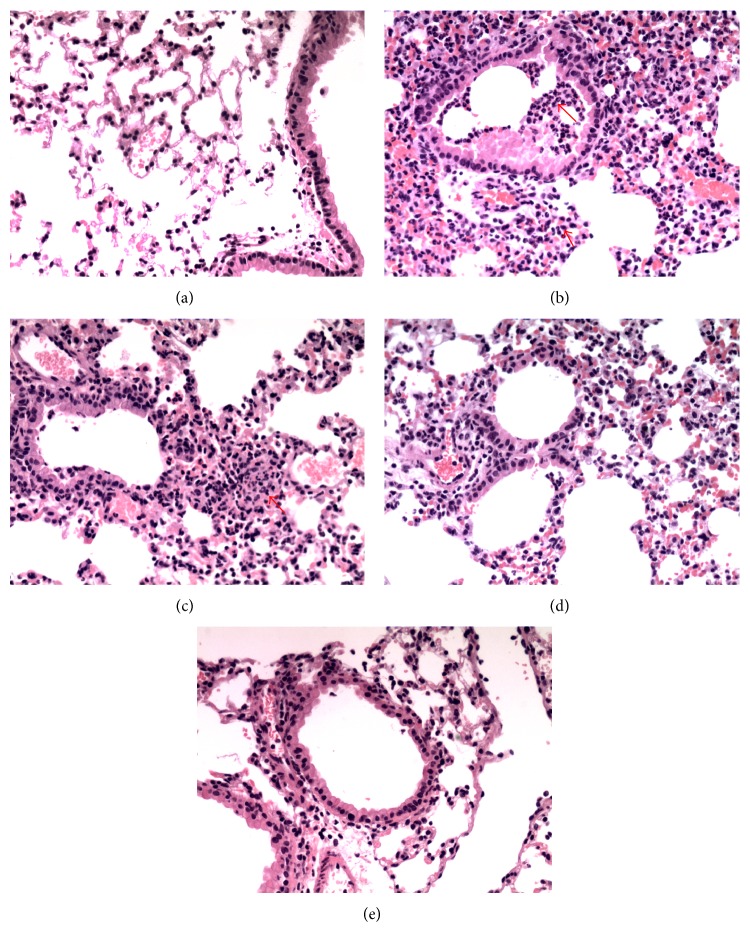
Effect of MAG on histopathological changes in lung tissues in LPS-induced ALI mice (light microscopy, ×200). MAG (3, 10, and 30 mg/kg) was injected intraperitoneally 1 h prior to LPS instillation. Lungs from each group were collected for histological evaluation at 24 h after LPS was given. Representative histological changes of lung obtained from mice of different groups. Control group (a) shows the normal parenchyma and lung airway; LPS group (b) shows tissue injury and neutrophil infiltration in the bronchovascular area (see arrows); LPS + MAG (3, 10, and 30 mg/kg) group (c, d, e) exhibit reduced tissue injury and neutrophil infiltration.

The lungs were harvested at 24 h after LPS injection and subjected to hematoxylin and eosin staining. The control group showed a normal structure. On the contrary, LPS group indicated significant histopathological changes, such as inflammatory cells infiltration, widespread alveolar wall thickness, and alveolar hemorrhage (Figure 3). However, LPS-induced histopathological changes were attenuated by MAG.

### 3.3. Effect of MAG on the Inflammatory Cell Counts in BALF of Mice with ALI

**Figure 4:**
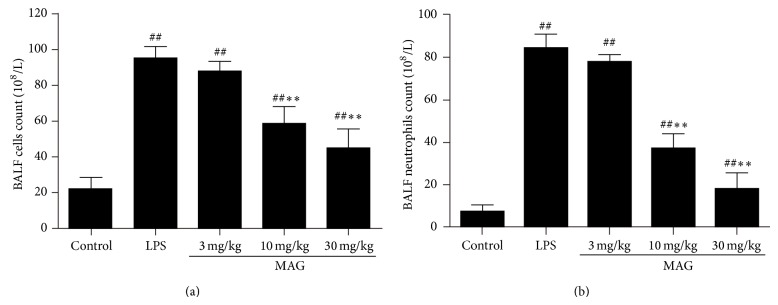
Effect of MAG on the number of total cells (a) and neutrophils (b) in the BALF of LPS-induced ALI mice. MAG (3, 10, and 30 mg/kg) was injected intraperitoneally 1 h prior to LPS instillation. The BALF was collected to measure the number of total cells (a) and neutrophils (b) at 24 h after LPS was given. The values presented are the mean ± SD. ^#^ *P* < 0.05, ^##^ *P* < 0.01 versus the control group; ^*^ *P* < 0.05, ^**^ *P* < 0.01 versus the LPS group.

Lipopolysaccharide challenge significantly increased the number of total cells and neutrophils, compared with the control group (*P* < 0.01) (Figure 4). The number of total cells and neutrophils in BALF was remarkably decreased via pretreatment with high and medium dose of MAG (10 and 30 mg/kg) (*P* < 0.01 and *P* < 0.01, resp.), while the low dose of MAG (3 mg/kg) did not decrease the number of total cells and neutrophils (*P* > 0.05).

### 3.4. Effect of MAG on the Concentrations of TNF-*α* and IL-1*β* in BALF of Mice with ALI

**Figure 5:**
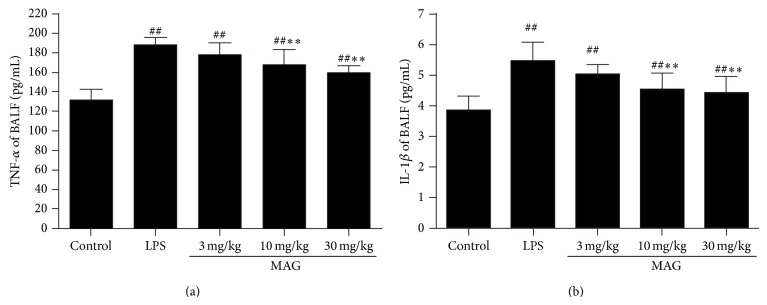
Effects of MAG on the production of inflammatory cytokines TNF-*α* (a) and IL-1*β* (b) in the BALF of LPS-induced ALI mice. MAG (3, 10, and 30 mg/kg) was injected intraperitoneally 1 h prior to LPS instillation. The BALF was collected at 24 h after LPS was given to analyze the inflammatory cytokines TNF-*α* (a) and IL-1*β* (b). The values presented are the mean ± SD. ^#^ *P* < 0.05, ^##^ *P* < 0.01 versus the control; ^*^ *P* < 0.05, ^**^ *P* < 0.01 versus the LPS group.

To determine the effects of MAG on LPS-induced cytokine production, we investigated inflammatory cytokines in the BALF. The concentrations of TNF-*α* and IL-1*β* in BALF were increased in LPS group, compared with the control group (*P* < 0.01) (Figure 5). Pretreatment with MAG (10 and 30 mg/kg) efficiently reduced the production of TNF-*α* and IL-1*β* (*P* < 0.01 and *P* < 0.01, resp.).

### 3.5. Effect of MAG on NF-*κ*B Activation of Mice with ALI

**Figure 6:**
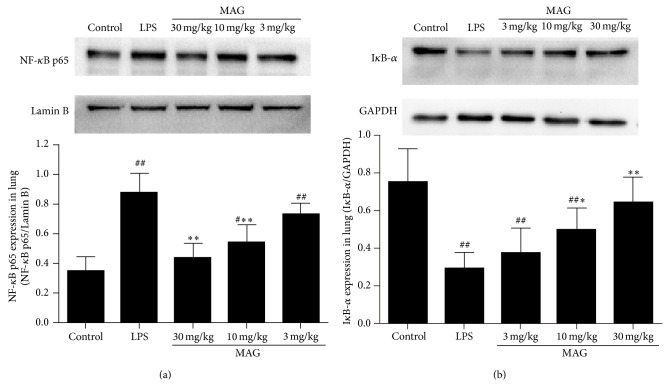
Effect of MAG on the activation of NF-*κ*B in the lungs of LPS-induced ALI mice. MAG (3, 10, and 30 mg/kg) was injected intraperitoneally 1 h prior to LPS instillation. Protein samples were analyzed by Western blot with specific antibodies as described. GAPDH and Lamin B were used as internal control. Similar results were obtained in at least three independent experiments. The values presented are the mean ± SD. ^#^ *P* < 0.05, ^##^ *P* < 0.01 versus the control group; ^*^ *P* < 0.05, ^**^ *P* < 0.01 versus the LPS group.

In order to assess the anti-inflammatory mechanism of MAG in LPS-induced ALI, we investigated the effect of MAG on NF-*κ*B activation in lung tissues. Nuclear Factor-Kappa B p65 expression was higher in LPS group than in the control group (*P* < 0.01), whereas MAG (10, 30 mg/kg) significantly decreased NF-*κ*B p65 protein expression, compared with LPS (*P* < 0.01 and *P* < 0.01, resp.) (Figure 6(a)). On the contrary, LPS significantly reduced I*κ*B-*α* protein expression compared with the control group (*P* < 0.01), whereas MAG (10 and 30 mg/kg) significantly increased I*κ*B-*α* expression, compared with the LPS group (*P* < 0.05 and *P* < 0.01, resp.) (Figure 6(b)).

## 4. Discussion

Known as a structural component of the outer membrane of gram-negative bacteria, LPS is capable of inducing ALI [14]. Lipopolysaccharide-induced ALI was characterized by alveolar epithelial permeability, lung edema, extensive infiltration of neutrophils, and release of inflammatory mediators [15]. Monoammonium glycyrrhizinate, a compound purified from licorice root, is often used as a sweetening and flavoring agent. Monoammonium glycyrrhizinate has a similarity with glycyrrhizin. Glycyrrhizin has been reported to decrease IFN-*γ*, IL-12, and IL-17 and increase IL-10, ameliorating TNBS-induced colitis in mice [16]. Glycyrrhizin was also reported to be able to attenuate lung inflammation by suppression of COX-2 and iNOS expression [17], upregulation of ICAM-1 and P-selectin expression, and formation of PAR and nitrotyrosine [18]. In this study, we investigated whether the anti-inflammatory effects of MAG on mice with ALI are through regulating NF-*κ*B signaling pathway.

We found MAG attenuated histopathological changes in the lung of LPS-pretreated mice. The results showed MAG significantly decreased the infiltration of inflammatory cells, especially the neutrophils. Monoammonium glycyrrhizinate also significantly reduced the W/D weight ratio of lungs. Additionally, MAG inhibited the production of TNF-*α*, IL-1*β*, and protein in the BALF. Furthermore, Western blotting showed that MAG inhibited the activation of NF-*κ*B. These results indicated the protective effects of MAG on the LPS-induced ALI in mice may be through inhibiting NF-*κ*B signaling pathway.

Lung histopathological changes occurred in mice of LPS group, including thickening of the alveolar wall, inflammatory cell infiltration, pulmonary congestion, and even lung tissue destruction. Compared with the LPS group, lung histopathological changes induced by LPS were decreased in MAG-pretreated group. Edema is another typical sign of the inflammatory response in LPS-induced ALI [4]. Endothelial injuries with microvascular leakage are considered the primary contributors of pulmonary edema. W/D weight ratio of the lung is a typical parameter to reflect the magnitude of pulmonary edema. Our study found that MAG significantly inhibited edema of the lung, as shown by the lower W/D weight ratio in the MAG (10 and 30 mg/kg) groups than in the LPS group. As we know, in the model of LPS-induced ALI, widespread destruction of alveolar epithelium makes proteinaceous exudates accumulate in the alveoli, representing the typical lesion in ALI [19]. We also found that in mice pretreated with high and medium dose MAG (10 and 30 mg/kg), protein extravasation was remarkably reduced compared to ALI mice. All those indicated the therapeutic effects of MAG on ALI.

Neutrophils played a critical role in ALI [20]. After intratracheal LPS instillation, neutrophils migrated across the endothelium and epithelium into the alveolar space and then they were activated, causing the excessive production of oxygen radicals and the release of granular enzymes, cytotoxic, and proinflammatory mediators [21, 22]. The result is a cascade-like response and tissue damage [23]. Neutrophils could release proinflammatory cytokines, like IL-1*β*, TNF-*α*, and IL-8. And the continuous existence of retained neutrophils had a close link with acute lung injury [24]. We found the number of total cells and neutrophils was markedly decreased in the MAG high and medium dose (10 and 30 mg/kg) groups, which was in agreement with the result of TNF-*α* and IL-1*β* below. But there was no significant difference between MAG low dose (3 mg/kg) group and LPS group.

Tumor necrosis factor-*α* (TNF-*α*) and interleukin-1*β* (IL-1*β*), as proinflammatory cytokines, play critical roles in ALI [25–27]. The increased levels of TNF-*α* and IL-1*β* were found in both ALI animals and ARDS patients. So proinflammatory cytokines, TNF-*α* and IL-1*β*, are vitally important in ALI [28]. Tumor necrosis factor-*α* could initiate and amplify the inflammatory cascade and contribute to the severity of lung injury in ALI [25]. Inhibiting TNF-*α* was reported to protect against ALI in mice [29]. What is more, TNF-*α* and IL-1*β* can stimulate production of a lot of other cytokines [30, 31]. These cytokines not only cause inflammatory injury, but also stimulate neutrophils to migrate into lung tissues [28], resulting in damage to lung. Besides, increased IL-1*β* had a profound influence on poor prognosis of ALI/ARDS patients. Our data showed that after LPS administration, the concentrations of TNF-*α* and IL-1*β* in BALF were higher compared with the control group; high and medium dose MAG (10 and 30 mg/kg) could inhibit the production of TNF-*α* and IL-1*β* induced by LPS. These results indicate that the protective effects of MAG on mice with ALI may be attributed to the inhibition of inflammatory cytokines.

Nuclear Factor-Kappa B is an important transcription factor and a regulator of many genes involved in acute lung injury [32]. It is well known that NF-*κ*B is a key signaling pathway accounting for the expressions of proinflammatory cytokines induced by LPS, such as TNF-*α* and IL-1*β* [33]. In animal models of ALI, NF-*κ*B is activated [34, 35] with increased TNF-*α* and IL-1*β*. At the same time, activated NF-*κ*B can recruit neutrophils into lung via CD11b/CD18 interaction with ICAM-1 [36]. Inhibition of NF-*κ*B pathway could decrease production of TNF-*α* and IL-1*β*, attenuate the neutrophil influx to the lung, and protect against ALI in animals [36]. Nuclear Factor-Kappa B p65 protein, an important transcriptional activator, is normally retained in the cytoplasm in an inactive form through being associated with inhibitory I*κ*B*α* [37]. Once activated by a wide variety of stimuli, I*κ*B inhibitor dissociated and NF-*κ*B p65 dissociates from its inhibitory protein I*κ*B-*α* and then translocates from the cytoplasm to the nucleus. In nucleus, NF-*κ*B p65 attaches to *κ*B binding sites and triggers the transcription of target genes such as TNF-*α* and IL-1*β* [38, 39]. There is no direct report about MAG and NF-*κ*B signaling pathway. We examined the effect of MAG on the activation of the NF-*κ*B signaling pathway to characterize the protective effects of MAG on acute lung injury induced by LPS. In the present study, LPS stimulation caused a significant increase in NF-*κ*B p65 protein expression and I*κ*B-*α* protein degradation, compared with the control group. Pretreatment with MAG could decrease the degradation of I*κ*B-*α* and nuclear translocation of NF-*κ*B p65. These results demonstrate that pharmacological inhibition by MAG exerts a protective effect on ALI via inhibiting NF-*κ*B-mediated inflammatory response.

## 5. Conclusions

Our findings showed that MAG could attenuate lung histopathological changes, reduce wet/dry weight ratio of the lung, and inhibit protein extravasation into alveolar space. The protective effects of MAG on ALI were correlated with the ability of reducing neutrophil infiltration and the production of TNF-*α* and IL-1*β* by suppressing the activation of NF-*κ*B signaling pathway. This indicates that MAG may be an agent for preventing and treating ALI.
